# Increased Protease-Activated Receptor-2 (PAR-2) Expression on CD14^++^CD16^+^ Peripheral Blood Monocytes of Patients with Severe Asthma

**DOI:** 10.1371/journal.pone.0144500

**Published:** 2015-12-14

**Authors:** Nami Shrestha Palikhe, Drew Nahirney, Cheryl Laratta, Vivek Dipak Gandhi, Dilini Vethanayagam, Mohit Bhutani, Irvin Mayers, Lisa Cameron, Harissios Vliagoftis

**Affiliations:** 1 Division of Pulmonary Medicine, Department of Medicine, University of Alberta, Edmonton, Alberta, Canada; 2 Alberta Asthma Center, University of Alberta, Edmonton, Alberta, Canada; 3 Department of Pathology, Schulich School of Medicine & Dentistry, Western University, London, ON, Canada; COCHIN INSTITUTE, Institut National de la Santé et de la Recherche Médicale, FRANCE

## Abstract

**Background:**

Protease-Activated Receptor-2 (PAR-2), a G protein coupled receptor activated by serine proteases, is widely expressed in humans and is involved in inflammation. PAR-2 activation in the airways plays an important role in the development of allergic airway inflammation. PAR-2 expression is known to be upregulated in the epithelium of asthmatic subjects, but its expression on immune and inflammatory cells in patients with asthma has not been studied.

**Methods:**

We recruited 12 severe and 24 mild/moderate asthmatics from the University of Alberta Hospital Asthma Clinics and collected baseline demographic information, medication use and parameters of asthma severity. PAR-2 expression on blood inflammatory cells was analyzed by flow cytometry.

**Results:**

Subjects with severe asthma had higher PAR-2 expression on CD14^++^CD16^+^ monocytes (intermediate monocytes) and also higher percentage of CD14^++^CD16^+^PAR-2^+^ monocytes (intermediate monocytes expressing PAR-2) in blood compared to subjects with mild/moderate asthma. Receiver operating characteristics (ROC) curve analysis showed that the percent of CD14^++^CD16^+^PAR-2^+^ in peripheral blood was able to discriminate between patients with severe and those with mild/moderate asthma with high sensitivity and specificity. In addition, among the whole populations, subjects with a history of asthma exacerbations over the last year had higher percent of CD14^++^CD16^+^ PAR-2^+^ cells in peripheral blood compared to subjects without exacerbations.

**Conclusions:**

PAR-2 expression is increased on CD14^++^CD16^+^ monocytes in the peripheral blood of subjects with severe asthma and may be a biomarker of asthma severity. Our data suggest that PAR-2 -mediated activation of CD14^++^CD16^+^ monocytes may play a role in the pathogenesis of severe asthma.

## Introduction

Severe asthma accounts for less than 10% of patients with asthma, but it is responsible for a large part of the health care cost associated with asthma [1]. The pathophysiological differences between severe and mild/moderate asthma are not well understood and no biomarkers have been identified that can reliably differentiate between these populations. Identifying pathophysiological pathways that are more active in patients with severe asthma could improve our understanding of the mechanisms of severe asthma, and provide targets for therapeutic interventions.

Severe asthma has been associated with eosinophilic [2] and/or neutrophilic inflammation [3], increased numbers and activity of Th2 cells [4] and mast cell activation [5] but there is little information regarding the role of monocytes in severe asthma. However, blood monocyte-derived macrophages from patients with severe asthma show reduced phagocytosis of *Haemophilus influenza* and *Staphylococcus aureus*, an observation that may indicate that patients with severe asthma are unable to eliminate bacteria from the airways, thus predisposing them to asthma exacerbations [6].

Circulating human monocytes are heterogeneous in morphology, phenotype and function [7]. Monocytes have been classified into 3 groups depending on their expression of the lipopolysaccharides (LPS) co-receptor CD14 and the Fcγ receptor CD16 [8]; CD14^++^CD16^-^ are classical monocytes, CD14^+^CD16^++^ are non-classical monocytes and CD14^++^CD16^+^ are intermediate monocytes. The frequency of CD14^++^CD16^+^ monocytes is increased in rheumatoid arthritis [9] and the frequency of both CD14^+^CD16^++^ and CD14^++^CD16^+^ is increased in type 2 diabetes [10] and sarcoidosis [11]. CD14^++^CD16^+^ monocytes have also been reported to be increased in patients with severe asthma compared to mild/moderate asthmatics [12]. However, the exact functional role of this monocyte subset in asthma and other inflammatory diseases is not known.

Protease-Activated Receptor-2 (PAR-2) belongs to a family of 7-transmembrane G protein-coupled receptors activated by serine proteases [13] involved in many inflammatory conditions. PAR-2 has been implicated in asthma through *in vivo* animal studies and *in vitro* human studies. We have recently shown, using mouse models of asthma, that PAR-2 plays an important role in the development of allergic sensitization to environmental aeroallergens [14, 15], but is also crucial for the development of allergic inflammation in sensitized mice ([16] and unpublished observations). PAR-2 can be activated by aeroallergens with serine protease activity and by endogenous serine proteases and PAR-2 expression is higher on the airway epithelium of patients with asthma [17]. However, little else is known regarding the role of PAR-2 in asthma in humans and there is no information about its potential role in the pathogenesis of severe asthma.

Many immune cells express PAR-2. Human monocytes express PAR-2 and upon PAR-2-mediated stimulation produce Interleukins (ILs) such as IL-1β, IL-6 and IL-8 [18]. However, there is no information regarding PAR-2 expression by immune and inflammatory cells in asthma.

Here we studied PAR-2 expression on peripheral blood inflammatory cells in patients with severe and mild/moderate asthma. We showed that there are increased numbers of CD14^++^CD16^+^ monocytes expressing PAR-2 in the peripheral blood of patients with severe asthma compared to patients with mild/moderate disease. We also showed that PAR-2 mRNA expression in whole blood correlates with airway function and total inhaled corticosteroid (ICS) dose. Finally, we showed that the % of CD14^++^CD16^+^PAR-2^+^ monocytes in peripheral blood were higher in those patients having asthma exacerbations over the last year compared to patients without exacerbations.

## Methods

### Subjects

We enrolled patients with severe (n = 12) and mild/moderate (n = 24) asthma as defined by the American Thoracic Society (ATS) [19]. The study was approved by the Ethics Committee, University of Alberta and all subjects gave written informed consent. Demographic data (age, sex, body mass index (BMI), and smoking history), atopic status (skin test reactivity to at least one of a panel of 12 aeroallergens), blood eosinophil numbers, serum Immunoglobulin E (IgE) levels, dose of oral corticosteroid (OCS), as well as history of OCS use, hospitalizations, and emergency department visits over the last year were collected.

### Analysis of PAR-2 expression by flow cytometry

Whole blood was obtained from subjects in heparinized tubes and differential counts were performed using Kimura staining as described [20]. Staining was performed on heparinized whole blood at room temperature. We used the following antibody panels for flow cytometry: 1. anti-PAR-2 antibody (SAM-11; Alexa Fluor 488, Santa Cruz Biotechnology), anti-CD14 (PerCP-Cy5.5; Ebiosciences), anti-CD16 (PE; BD Bioscience), and anti-CD4 (APC; BD Bioscience) and 2. anti-PAR-2 (SAM-11) and anti-CCR3 (PE; CD193, BD # 558165). Results were analyzed using FLOWJO (TreeStar, Ashland OR, USA). The following strategies were used to identify the various cell populations analyzed: *Monocytes*: Cells from the lymphocyte/monocyte gate were further analyzed by their expression of CD14 (PerCP-Cy5.5) to identify monocytes and CD16 (PE) to separate classical from intermediate and non-classical monocytes. Cells that were CD14^++^CD16^+^ were considered intermediate monocytes, whereas cells that were CD14^++^CD16^-^ were considered classical monocytes. *Lymphocytes*: We analyzed CD4^+^ T lymphocytes (cells in the lymphocyte/monocyte gate expressing high levels of CD4). *Eosinophils*: To identify eosinophils we looked at high SSC vs CCR3^+^ (PE) (CD193). These cells were then plotted against PAR-2 (488) and CCR3 (PE) to look for the expression of PAR-2 on eosinophils. *Neutrophils*: The neutrophils were gated by drawing a gate around all the granulocytes high SSC and high FSC. The granulocyte gate was then plotted against CD16 (PE) and PAR-2 (488), the double positives were used to determine the expression of PAR-2 on CD16^+^ granulocytes (neutrophils).

### Quantitative real time PCR (qRT-PCR)

Whole blood RNA was isolated from PAXgene whole blood RNA tubes (PreAnalytiX, Qiagen/BD) using the PAXgene Blood RNA kit (PreAnalytiX). *PAR-2* mRNA was quantified using a custom FAM-labeled DTAM probe (5’-TAA GGT TGA TGG CAC ATC CCA CGT CAC TGG -3’) (Genosys probe) and the following primers *PAR-2-*F (5’-TGC TAG CAG CCT CTC TCT CC -3’) and *PAR-2*-R (5’-CCA GTG AGG ACA GAT GCA GA-3’). *GAPDH* mRNA was used as house keeping control and was quantified using a custom 6FAM-labeled TAMRA probe (5’-AAA TCC CAT CAC CAT CTT CCA GGA GCG A-3’) (Applied Biosystems) and the following primers *GAPDH-*F (5’-CTG AGA ACG GGA AGC TTG TCA -3’) and *GAPDH*-R (5’-GCA AAT GAG CCC CAG CCT T-3’). The PCR protocol consisted of 10 minutes at 95°C followed by 40 cycles of 30 seconds at 95°C and 60 seconds at 60°C. Samples were run in triplicate. Data were calculated and represented as “*PAR-2* copies per 1000 copies of *GAPDH”*.

CRTh2 (Hs00173717_ml) was amplified by TaqMan assay (Applied Biosystems, Carlsbad, CA, USA). CRTh2 mRNA was quantified using the delta-delta Cycle threshold (ddCt) method.

### Statistical Analysis

Difference in the mean value of phenotypic clinical characteristics between study subjects was compared using the Mann-Whitney U-test for continuous variables and Fisher-exact t-test was used for categorical variables. For receiver operating characteristics (ROC) curves and area-under-curve (AUC), 95% confidence intervals were used. Statistical analyses were performed using SPSS (version 21.1, Chicago, IL, USA) and optimum cut offs were determined using R software (version 3.1.1, The R Foundation for Statistical Computing). Statistical significance was set at *P*<0.05.

## Results

### Demographics and clinical characteristics

The demographic characteristics of the two groups of patients with asthma are presented in Table 1. No significant differences were observed in mean age, sex, BMI, atopic status, peripheral blood eosinophil numbers, total serum IgE and smoking status. Forced expiratory volume in 1 sec (FEV_1_) (% predicted) and FEV_1_/forced vital capacity (FVC) ratio were significantly lower in subjects with severe compared to mild/moderate asthma. All severe asthmatics, but only 62% of mild/moderate, were on daily ICS (*P* = 0.016). Patients with severe asthma were on a higher total daily dose of ICS compared to those with mild/moderate asthma (*P*<0.001).

**Table 1:** Demographic and clinical characteristic of study subjects.

Clinical demographics	Mild/Moderate asthma (n = 24)	Severe asthma (n = 12)	*P*
Mean Age (Min-Max) (y)	39.3±2.8 (22–68)	43.0±4.2 (28–67)	0.512
No of female patients (%)	14 (58.3%)	5 (41.7%)	0.483
BMI (kg/m^2^)	33.7±4.0	32.8±5.9	0.788
Atopy (%)*	18 (90%)	8 (72.7%)	0.317
Log transformed IgE (kU/L)**	1.9±0.1	2.0±0.2	0.615
Eos (cells/μl)***	326.1±53.4	200.0±42.6	0.138
FEV_1_ (% predicted)	85.8±2.5	68.0±5.6	**0.009**
FEV_1_/FVC (%)	72.1±2.2	57.6±3.5	**0.002**
FEV_1_<80% predicted	9 (37.5%)	9 (75%)	0.075
History of Smoking (%)	9 (37.5%)	5 (41.7%)	1.000
Mean Pack Years	2.8±1.0	6.3±3.0	0.639
Current Smoking (%)	3 (12.5%)	0 (0%)	0.536
Daily 2^nd^ line controller	15 (62.5%)	12 (100%)	**0.016**
Total ICS dose (fluticasone equivalent—μg/day)	410.5±71.4	1134.0±51.4	**<0.001**

Because of missing data points, “n” for the categories labeled *, ** and *** is not the total of the population, but the numbers shown below:

* n = 20 for mild/moderate and n = 11 for severe asthma;

** n = 17 for mild/moderate and n = 10 for severe asthma;

*** n = 23 for mild/moderate and n = 12 for severe asthma

### PAR-2 expression in CD14^++^CD16^+^ monocytes was higher in severe compared to mild/moderate asthma

**Fig 1:**
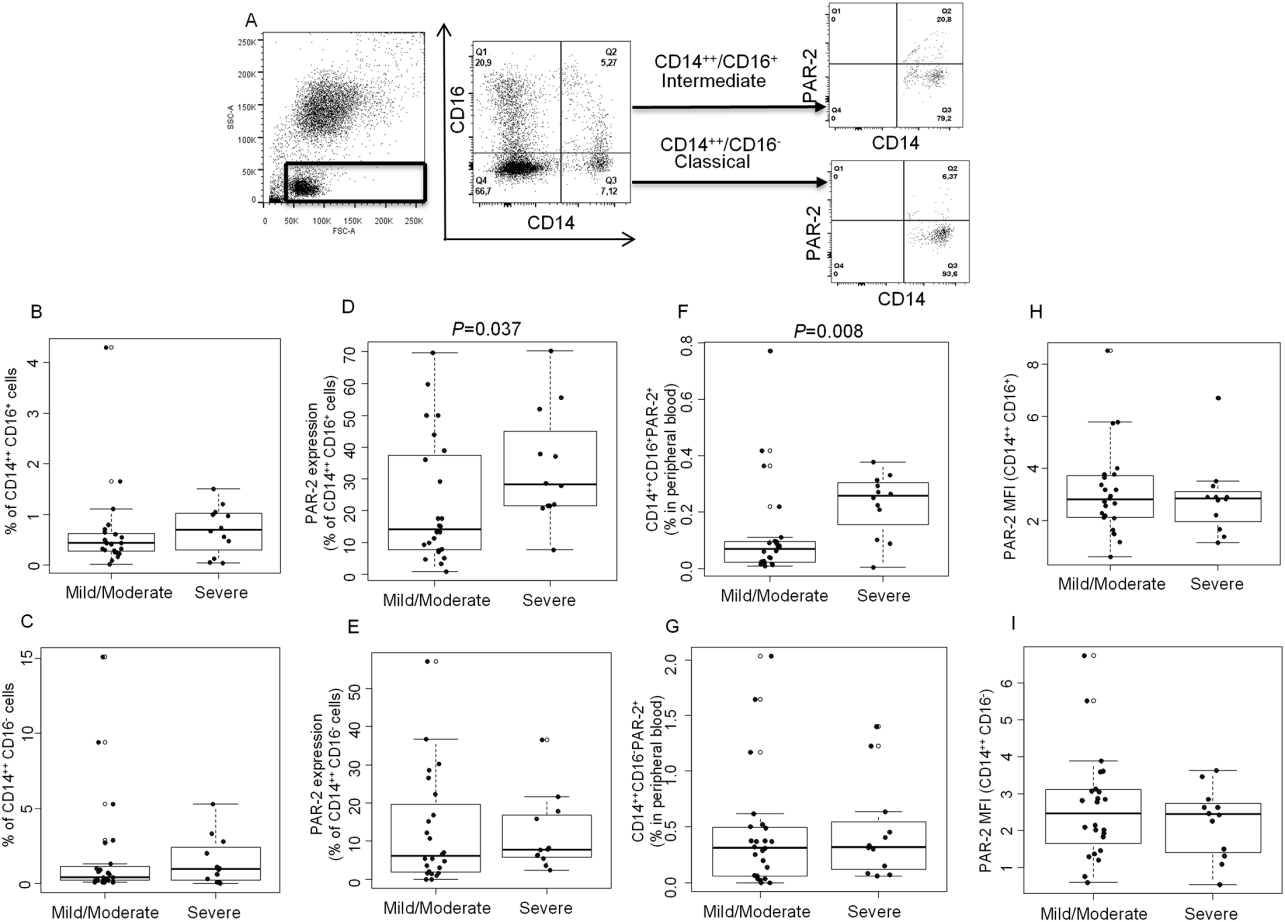
PAR-2 expression on monocytes and severe asthma **A.** Gating strategy to study PAR-2 expression on peripheral blood monocytes. B-C. Percentage of CD14^++^CD16^+^ (B) and CD14^++^CD16^-^ (C) monocytes in the peripheral blood of severe asthmatics compared to mild/moderate asthmatics. D-E. PAR-2 expression on CD14^++^CD16^+^ (D) and CD14^++^CD16^-^ (E) monocytes in patients with mild/moderate and severe asthma. F-G. Percentage of CD14^++^CD16^+^PAR-2^+^ (F) and CD14^++^CD16^-^PAR-2^+^ (G) monocytes in peripheral blood of patients with mild/moderate and severe asthma. H-I. PAR-2 MFI on CD14^++^CD16^+^ (H) and CD14^++^CD16^-^ (I) monocytes from patients with mild/moderate and severe asthma. Data is presented as boxplots (n = 24 for mild/moderate and n = 12 for severe asthma). Statistical significance was assessed by Mann-Whitney rank sum test, with *P*<0.05 considered significant.

Monocyte subsets were defined as CD14^++^CD16^-^ (classical) and CD14^++^CD16^+^ (intermediate) within the lymphocyte/monocyte gate in side scatter/forward scatter plots. These subsets were then analyzed for PAR-2 expression separately (Fig 1A). There was no difference in percentage of CD14^++^CD16^+^ (Fig 1B) and CD14^++^CD16^-^ (Fig 1C) monocytes in the peripheral blood of severe asthmatics compared to mild/moderate asthmatics. Analysis of PAR-2 expression on monocyte subsets showed that a significantly higher percentage of CD14^++^CD16^+^ monocytes expressed PAR-2 in severe asthmatics compared to mild/moderate asthmatics (33.59±5.15 vs. 22.40±4.03, *P* = 0.037) (Fig 1D), but there was no difference between the two populations in the % of CD14^++^CD16^-^ monocytes expressing PAR-2 (11.66±2.82 vs. 12.53±2.93) (Fig 1E). Severe asthmatics had also higher percent of CD14^++^CD16^+^PAR-2^+^ cells (intermediate monocytes expressing PAR-2) in peripheral blood compared to mild/moderate asthmatics (0.22±0.03 vs. 0.11±0.03, *P* = 0.008) (Fig 1F), but the percent of CD14^++^CD16^-^PAR-2^+^ monocytes in the two groups was similar (Fig 1G). There was no difference in PAR-2 mean fluorescent intensity (MFI) between severe and mild/moderate asthmatics on any of the two monocyte subsets (Fig 1H and 1I).

**Fig 2:**
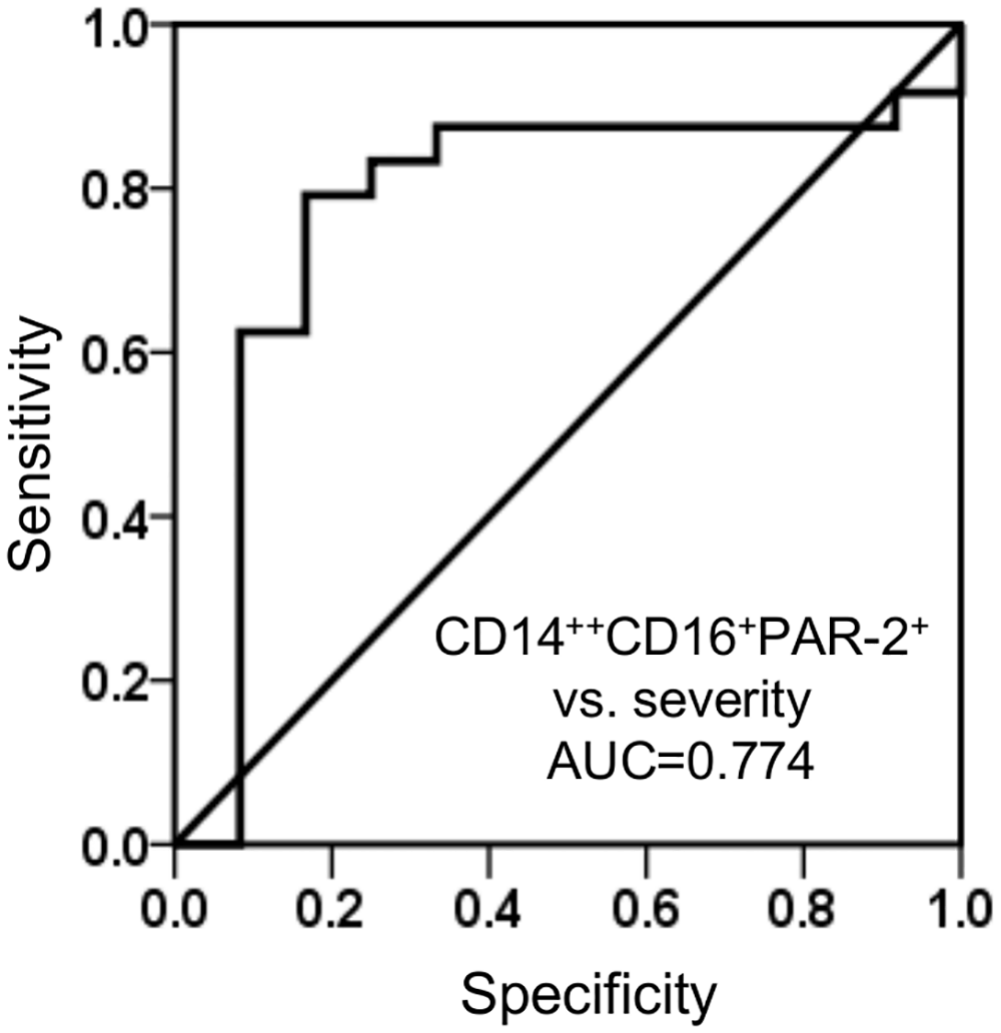
ROC curve of “% of CD14^++^CD16^+^PAR-2^+^ monocytes” in peripheral blood used to identify patients with severe asthma. Sensitivity and specificity were calculated according to the optimal cutoffs using R software and AUC with a 95% confidence interval is shown.

ROC curve analysis showed that the percent of CD14^++^CD16^+^PAR-2^+^ in peripheral blood was able to discriminate between patients with severe and those with mild/moderate asthma with high sensitivity and specificity (AUC = 0.774, *P* = 0.008, 95% CI, sensitivity of 83.3% and specificity of 79.2%) (Fig 2).

We also analyzed PAR-2 expression on eosinophils (CCR3^+^SSC^high^ cells), neutrophils (CD16^+^SSC^high^ cells) and CD4^+^ lymphocytes (CD4^+^ cells in lymphocyte gate). Low percentages of cells of each of these cell types expressed PAR-2 and there were no differences in expression between the two patient groups (data not shown).

### PAR-2 mRNA expression in whole blood correlates with airway obstruction and daily inhaled corticosteroid dose

**Fig 3:**
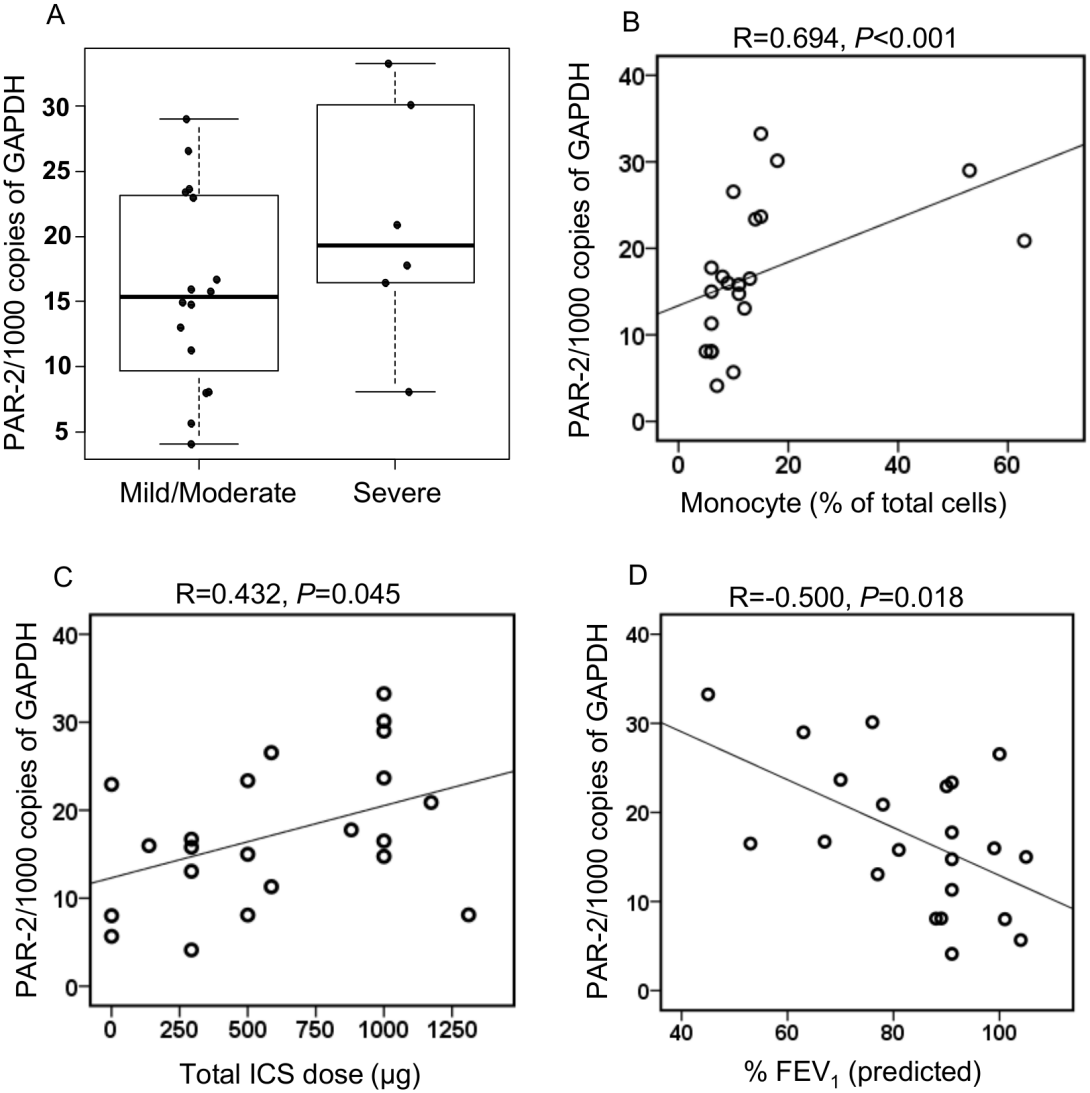
PAR-2 mRNA expression in whole blood of patients with asthma. (A) PAR-2 mRNA expression in mild/moderate (n = 16) and severe (n = 6) asthmatics. (B) correlation of PAR-2 mRNA expression with percentage of monocytes in peripheral blood. (C) total ICS dose and (D) percentage of FEV_1_ predicted in the whole population (n = 22).

We also performed qRT-PCR analysis of PAR-2 mRNA expression using RNA isolated from whole blood of the study subjects. There was no difference in the levels of PAR-2 mRNA, expressed as copies of PAR-2/1000 copies of GAPDH between severe and mild moderate asthmatics (21.10±3.78 vs. 15.87±1.88) (Fig 3A). However, PAR-2 mRNA was strongly correlated with monocyte count by Kimura staining in the whole population (R = 0.694, *P*<0.001) (Fig 3B), but not with counts of other cells (data not shown)

PAR-2 mRNA expression in the whole population was also positively correlated with the total daily dose of inhaled corticosteroids (R = 0.432, *P* = 0.045) (Fig 3C), and negatively correlated with FEV_1_ (% predicted) (R = -0.500, *P* = 0.018) (Fig 3D), but was not correlated with serum IgE (data not shown).

Interestingly PAR-2 mRNA level was also correlated with CRTh2 mRNA in whole blood (R = 0.579, *P* = 0.005), and with the percent of CD4^+^ T cells expressing CRTh2 (R = 0.482, *P* = 0.023), indicating that PAR-2 mRNA expression correlates with the degree of Th2 inflammation.

### Asthma exacerbations and PAR-2 expression

**Fig 4:**
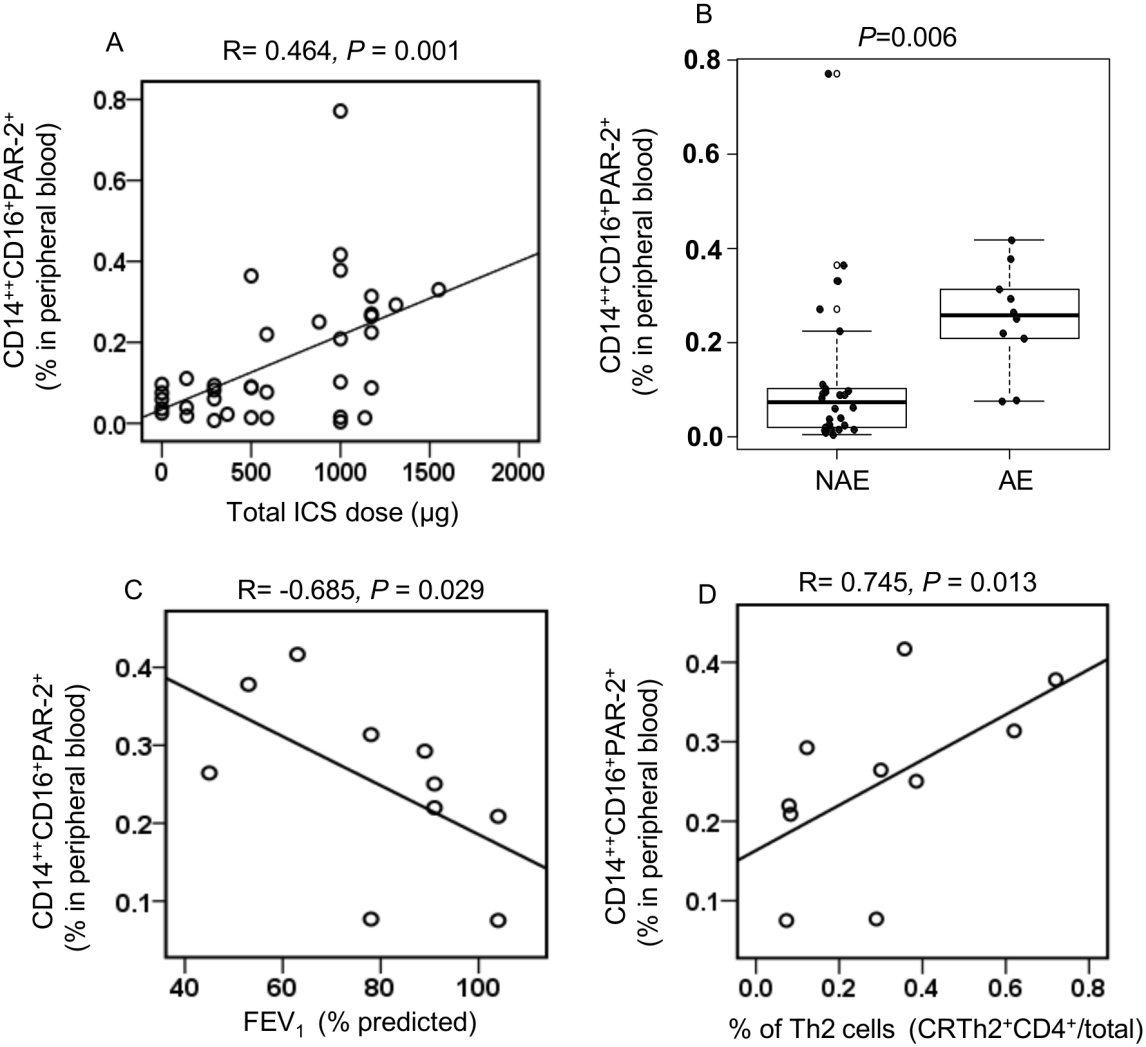
Asthma exacerbations and CD14^++^CD16^+^PAR-2^+^ expression. (A) Proportion of CD14^++^CD16^+^PAR-2^+^ cells in peripheral blood was correlated with the total daily dose of ICS in the whole population. (B) Percentage of CD14^++^CD16^+^PAR-2^+^ monocytes in peripheral blood in asthmatics that did not have recent exacerbations (NAE) (n = 26) compared to asthmatics with recent exacerbations (AE) (n = 10). Correlation of the “% of CD14^++^CD16^+^PAR-2^+^ monocytes in peripheral blood” (C) percentage predicted FEV_1_ and (D) “% of CRTh2^+^CD4^+^” in peripheral blood in subjects with asthma exacerbations (n = 10).

We showed that PAR-2 expression on CD14^++^CD16^+^ monocytes are increased in patients with severe asthma. We then analyzed further our data for clinical characteristics that may explain the difference in PAR-2 expression by CD14^++^CD16^+^ monocytes in the two study populations. Patients with severe asthma were on higher total daily dose of inhaled corticosteroids compared to patients with mild/moderate disease (Table 1). We therefore, analyzed our data to test whether PAR-2 expression was correlated with total daily dose of inhaled corticosteroids. The proportion of CD14^++^CD16^+^PAR-2^+^ cells in peripheral blood was correlated with the total daily dose of ICS in the whole population (R = 0.464, *P* = 0.001), (Fig 4A) but not with the total daily dose of ICS in each one of the patient groups separately (data not shown). We also noticed that 50% of the severe asthma subjects in our study had asthma exacerbations over the last year. We therefore asked the question whether the difference in PAR-2 expression between monocytes from severe versus mild/moderate asthmatics was the result of disease severity or a marker of frequent exacerbations. To do this we separated our whole study population into two groups, patients that did not have a recent exacerbation (NAE) and those with recent exacerbations (AE). In accordance with Fajt et al [4] we defined patients with a AE as those reporting 3 or more OCS bursts, asthma-related urgent care visits, or hospitalization in the 12 months prior to recruitment. There was higher percentage of CD14^++^CD16^+^PAR-2^+^ cells (0.25±0.03 vs 0.11±0.03, *P* = 0.006) in peripheral blood of asthmatics with asthma exacerbations compared to the rest of the population (Fig 4B).

Interestingly, % of CD14^++^CD16^+^PAR-2^+^ in peripheral blood was negatively correlated with pulmonary lung function, FEV_1_ (% predicted) (R = -0.685, *P* = 0.029 Fig 4C) and positively correlated with the percent of CD4^+^ T cells in peripheral blood expressing CRTh2 (R = 0.745, *P* = 0.013, Fig 4D) in the group of patients having asthma exacerbations.

## Discussion

Our study showed that subjects with severe asthma had higher percent of CD14^++^CD16^+^ cells expressing PAR-2 compared to patients with mild/moderate asthma, indicating that PAR-2-expressing CD14^++^CD16^+^ monocytes may be involved in the pathogenesis of severe asthma. This is the first report showing a correlation of PAR-2 expression on a peripheral blood monocyte subset with asthma severity. In contrast, peripheral blood eosinophils, neutrophils and CD4^+^T cells had very low levels of PAR-2 expression and there was no difference in the level of expression between cells from severe asthmatics and cells from patients with mild/moderate asthma. Furthermore, ROC curve analysis showed that the percent of CD14^++^CD16^+^PAR2^+^ in peripheral blood, with a cut-off point of 0.21%, discriminates severe asthmatics from mild/moderate asthmatics with high sensitivity and specificity. This result indicates that PAR-2 expression on peripheral blood intermediate monocytes may be a useful biomarker to identify patients with severe asthma.

CD14^++^CD16^+^ monocyte numbers are increased in various inflammatory conditions including rheumatoid arthritis [9] and sarcoidosis [21]. A previous report suggests that the numbers of CD14^++^CD16^+^ monocytes are increased in patients with severe asthma compared to patients with mild and those with moderate asthma [12]. In our study we did not find such a difference. The reason for this discrepancy is not clear except to say that the two studies have used different flow cytometry gating strategy to identify monocyte subgroups and the severe asthma population in the previous study had lower FEV_1_ than the severe asthma population in our study indicating that the previous study may have included patients with more severe disease.

The level of PAR-2 expression on blood cells in our study is in line with a previous report indicating that up to 40% of peripheral blood monocytes but less than 4% of CD4^+^ lymphocytes express PAR-2 [22]. PAR-2 expression on monocytes of patients with rheumatoid arthritis [23], granulomatosis with polyangiitis [24] and primary antiphospholipid syndrome [25] correlates with disease activity. However, to our knowledge, this is the first study to describe PAR-2 expression on specific monocyte subgroups and to correlate this expression with disease severity in a chronic inflammatory condition.

Although PAR-2 expression on monocytes was described more than 10 years ago [26] the biological function of PAR-2 expressed on monocytes remains poorly understood. PAR-2-mediated activation of peripheral blood monocytes leads to release of inflammatory mediators, [18], increases phagocytosis of *Staphylococcus aureus* and *Escherichia coli* [27] and enhances IFNγ-dependent suppression of influenza A replication [28]. These observations indicate that PAR-2 activation of monocytes may act independently or synergistically with other immune pathways to enhance immunity against infectious agents, but also to propagate inflammation. Further work is needed to better understand the role of PAR-2-mediated monocyte activation in inflammatory diseases.

Since the function of CD14^++^CD16^+^ monocytes in asthma or other chronic inflammatory conditions is not known, the clinical significance of our observation of increased PAR-2 expression on CD14^++^CD16^+^ monocytes of patients with severe asthma is unclear. CD16^+^ monocytes preferentially express receptors for chemokines such as CCL3 and CCL5 [29]; these chemokines are increased in patients with asthma [30, 31], indicating a potential link between CD16^+^ monocytes and asthma pathophysiology. Severe asthma has also been linked to the presence of Th17-related cytokines [32], and CD14^++^CD16^+^ monocytes may mediate Th17 cell expansion [9]. Furthermore, CD14^++^CD16^+^ monocytes have high HLA-DR expression and facilitate antigen presentation [33] and can be pro-inflammatory through the release of TNF [34] and/or ROS [35]. These observations suggest that increased numbers of PAR-2 expressing CD14^++^CD16^+^ monocytes may be pathogenetic in severe asthma through many different pathways. Purification of CD14^++^CD16^+^ monocytes followed by studies on the biological consequences of *in vitro* PAR-2-medicated activation of these cells will clarify this issue.

We further analyzed our data to better understand the relationship between numbers of CD14^++^CD16^+^PAR2^+^ cells and asthma severity. Our severe asthmatic group was on higher total daily dose of ICS and experienced more exacerbations compared to the mild/moderate group. Therefore, there is the possibility that PAR-2 expression on CD14^++^CD16^+^ cells are influenced either by ICS or by asthma exacerbations. A previous study has demonstrated that glucocorticoid treatment is associated with an enrichment of CD14^++^CD16^+^cells in patients with autoimmune uveitis [36]. However, in our study we did not observe a correlation between CD14^++^CD16^+^ cells numbers and total daily dose of ICS. We observed however, a positive correlation between total daily ICS dose and PAR-2 expression by CD14^++^CD16^+^ cells in our total population, but did not find the same association when we analyzed severe and mild/moderate asthmatics separately. The biological significance of this observation is not clear at this time. To understand whether frequent exacerbations are associated with PAR-2 expression on CD14^++^CD16^+^ monocytes we characterized our patients into groups depending on the presence of an exacerbation over the last year. Asthmatic patients with an exacerbation in the previous 12 months had higher percentage of CD14^++^CD16^+^ monocytes expressing PAR-2 in peripheral blood compared to subjects without an exacerbation. Whether this is the result of specific characteristics of patients with frequent exacerbations, or the fact that there were more patients with severe asthma in the recent exacerbation group, cannot be answered from our data. Analysis of a larger cohort and availability of data on asthma control and daily dose of ICS used at the time of measurement of PAR-2 expression on monocytes may allow us to clarify these questions. However, the fact that patients that experienced exacerbations over the last year had higher PAR-2 expression on CD14^++^CD16^+^ cells and their lung function inversely correlated with PAR-2 expression, indicates that the % CD14^++^CD16^+^PAR-2^+^ may be an important inflammatory biomarker for both severe asthma and for severe asthma with frequent exacerbations.

In this study, we also measured whole blood PAR-2 mRNA expression by qRT-PCR. We found that PAR-2 mRNA expression correlated with the total number of monocytes in blood (Fig 3B), but not with the numbers of other cell types. Monocytes therefore may be the main contributors to PAR-2 mRNA levels in whole blood. In contrast there was no difference in the PAR-2 mRNA expression in whole blood between severe and mild/moderate asthmatics indicating that PAR-2 expression on monocytes other than CD14^++^CD16^+^ monocytes may be the main driver of PAR-2 mRNA expression in whole blood. Interestingly, PAR-2 mRNA expression in whole blood was negatively correlated with FEV_1_ (% predicted) and positively correlated with two markers of Th2 inflammation, namely CRTh2 mRNA and percent of CRTh2^+^CD4^+^ T cells in peripheral blood. This observation suggests that PAR-2 activation may be associated with Th2 responses, although the mechanism for this association is not clear.

In conclusion, this study shows that PAR-2 expression is increased on inflammatory monocytes in two groups of patients with asthma; PAR-2 expression on inflammatory monocytes is increased in subjects with severe asthma and in subjects that have experienced asthma exacerbations over the last year irrespective of disease severity. This novel finding identifies a potential new biomarker for severe asthma. Larger studies will be needed to confirm the finding, and to understand the relevance of PAR-2 expression on subgroups of peripheral blood monocytes in asthma pathogenesis.

## Abbreviations

ATS: American Thoracic Society
AUC: Area under curve
BMI: Body mass index
CCL3: Chemokine (C-C motif) ligand 3
CCL5: Chemokine (C-C motif) ligand 5
FEV_1_: Forced expiratory volume in 1 sec
FVC: Forced vital capacity
ICS: Inhaled corticosteroid
IgE: Immunoglobulin E
ILs: Interleukins
LPS: Lipopolysaccharide
NAE: No recent exacerbation
OCS: Oral corticosteroids
PAR: Protease-activated receptor
qRT-PCR: Quantitative real-time PCR
RE: Recent Exacerbation
ROC: Receiver operating characteristics
